# Using Bonferroni, BIC and AIC to assess evidence for alternative biological pathways: covariate selection for the multilevel Embryo-Uterus model

**DOI:** 10.1186/1471-2288-13-73

**Published:** 2013-06-06

**Authors:** Christos Stylianou, Andrew Pickles, Stephen A Roberts

**Affiliations:** 1 Centre for Biostatistics, Institute of Population Health, University of Manchester, 1st Floor, Jean McFarlane Building, Manchester, M13 9PL, UK; 2 Institute of Psychiatry, King’s College, London, SE5 8AF, UK

**Keywords:** Embryo uterus models, In-vitro fertilization, Hypothesis testing, Model selection, Information criteria, Simulation

## Abstract

**Background:**

IVF treatments for infertility involve the transfer of multiple embryos in any one treatment cycle. When data is available on individual embryos the outcomes of each embryo are only partially observed, as treatment outcome (live birth) is assessed at the patient level. Two-level Embryo-Uterus (EU) models have been developed which assume a biologically plausible mechanism and assume that effects are mediated directly through the embryo (E) and also through the uterine environment (U), represented by two sub-models. This approach potentially allows inference as to the association of patient variables with outcome. However, when the variable is measured at the patient level either additional decisions have to be made in the modelling process as to in which sub-model the variable should be included or some model selection algorithm has to be invoked. These uncertainties have limited the practical application of these models.

**Methods:**

We have conducted simulation studies based around realistic parameter values of situations where a putative patient-level variable is being considered for inclusion in an EU model and/or the mechanistic interpretation from the sub-model assignment is of interest. Firstly we explore various strategies for inference for a variable of interest where the sub-model is either pre-specified or considered unknown. Secondly we explore the use of information criteria to select the appropriate sub-model and the strength of evidence for that assignment. These are demonstrated in a reanalysis of a previously published dataset.

**Results:**

In the absence of prior evidence for potential prognostic factors measured at the patient level, two single degree-of-freedom likelihood ratio tests with a Bonferroni correction including the variable of interest in first the E then the U sub-model performs well as a statistical test for association with outcome. For model building the information criteria can be used, but large differences are required (⪆6) to provide reasonable evidence of sub-model assignment. Previous interpretations have been over-optimistic.

**Conclusions:**

These results suggest simple strategies and should enable these models to be used more confidently in practical applications.

## Background

In-vitro Fertilization (IVF) is a treatment for infertility in which embryos are created outside of the prospective mother and, after culture for 3–6 days, one or more embryos are transferred to her uterus. Analysis of data arising from IVF treatment often includes prognostic factors observed at an embryo level. However the outcome at this embryo level is often only partially observed. This partial observability arises due to the fact that individual embryos cannot be tracked after transfer and, unless all or none of the transferred embryos develop, it is not possible to determine which embryo(s) implanted. Analysis has to either be conducted at the patient level using aggregated embryo data or non-standard methodology is required.

IVF treatments have a hierarchical structure with effects on the embryo from both mother and father and can also have a complex nested structure [1] if effects across multiple treatment cycles are included and donor eggs or sperm are utilised. Considering just the embryo transfer process in these treatments, Spiers *et al.*[2] suggested a model based on a plausible biological mechanism. These models are named “embryo-uterus” (EU) models and are based on the idea that the successful development of an embryo depends independently on two binary factors, the embryos own inherent viability (E) and the receptivity of the uterus (U). For an embryo to develop it must both be viable (E = 1) and placed in a receptive uterus (U = 1). Spiers *et al.*[2] assumed E and U had constant probabilities (*e* and *u*) over the whole population. In later work, by Baeten *et al.*[3] an EUL model was proposed, assuming the *e* probability was constant over the whole population and modelling U through logistic regression. A more general approach was developed by Zhou and Weinberg [4] which replaced both E and U with logistic regression sub-models allowing the inclusion of patient and embryo covariates. Although the original derivation assumed a very specific biological mechanism, the interpretation has evolved somewhat, and in particular it is acknowledged that the U component may be only partially related to uterine receptivity and can include contributions from a range of factors related to treatment [5,6]. These approaches have been utilised for predictive and inferential analyses in a number of practical applications for the assessment of prognostic factors [5-7] and developing predictive models for assessing alternative treatment pathways [8].

The EU model has an explicit multi-level structure with the transferred embryos nested within the uteri. A prognostic factor measured at the patient level, can validly be included in either the E or the U sub-models (or both) and therefore assumed or inferred to be operating through either the uterine receptivity of the patient or the viability of the embryos. Whilst it may seem counter-intuitive to model a U-level covariate at the E-level, the implied mechanism does suggest that this is what can be required, for instance donor egg data [9] suggests strongly that embryo viability declines with age whilst maternal receptivity does not decline as strongly. As noted by Roberts *et al.*[10] the level at which a covariate acts is identifiable (albeit not strongly) through the twin rate if there are multiple embryos transferred per cycle.

The potential to choose in which of the two sub-models a covariate should be included adds an extra level of complexity to the practical use of these models. The natural biological interpretation means that this choice is not merely a statistical convenience, but that identification of the appropriate level may have a biological interpretation with clinical consequences. For example, age has been shown to primarily affect the embryo with a much weaker effect on the uterine component and so the effects of ageing can be offset by the use on egg donation or preservation. The presence of a substantial uterine component means that the loss in pregnancy rates associated with a move from double to single embryo transfer is more modest than would otherwise be expected [11]. In practical applications some authors have avoided any statistically-based decision and instead made the choice of sub-model arbitrarily [7,10] or based on “previous knowledge” [5]. Roberts *et al.*[5] used the Akaiki Information Criterion (AIC) criterion comparing the model with the effect in the E and the model with the effect in the U. In this work the authors noted that while the AIC distinguished a “best” model, it was difficult to provide any clear statement as to the weight of evidence in favour of the particular chosen model and its biological interpretation. Although suggestions on the interpretation of BIC differences have been made previously in different contexts [12,13], it is not clear how or whether such suggestions have relevance here. Others have chosen the patient covariates to be considered for inclusion in the model by constructing two multivariate logistic regression models, a pregnancy model and a twin pregnancy model, using a backward stepwise elimination [7]. Subsequently a number of models were considered consisting of a number or all the covariates included in the final logistic models in either the E and U with the choice of the final model being made by the models predictive abilities for pregnancy and multiple pregnancy.

As these models are now being utilised for practical applications, there is a need for guidance on how inference and model selection should be performed. When using these models to determine whether a variable is associated with outcome (inference), unless the sub-model can be pre-specified, there will be explicit or implicit multiple hypotheses tests associated with each variable. Thus it will be generally anti-conservative to test the parameter only in the sub-model selected (by whatever procedure), or to perform two tests at the nominal level. On the other hand the non-independence of the tests suggests it may be over-conservative to test in both sub-models with a standard Bonferroni correction. When developing prognostic or predictive models an information criterion approach to model selection is attractive, particularly as some of the comparisons of interest are not naturally nested. However there is a need for guidance on how to interpret the information criteria in terms of the weight of evidence that an effect can be properly ascribed to the E or U component.

### Motivating example

This work arose out of issues encountered in the *toward*SET project, which involved the use of EU models on large datasets to predict outcomes for various options for the implementation of a single embryo transfer policy. Details of this dataset have been published elsewhere [5,11]. The main aim of the *toward*SET project was pragmatic, to develop a useable predictive model to allow modelling of potential treatment policies. However, given that the dataset was large and comprehensive, there was also interest in the prognostic model itself: which factors were predictive of outcome and in particular was their effect mediated through the embryo or the recipient mother. This particular analysis is described in [5]. The dataset comprised 12,487 fresh treatments from 8775 couples across 5 UK centres and included 16 categorical patient and treatment factors along with two measures of embryo quality.

### Outline of present work

This present work presents a series of simulation studies which aims to clarify the issues underpinning the use of EU models for practical applications. Firstly we assess the performance of alternative strategies for the inference problem with some consideration as to power and sample size issues. Secondly we evaluate the use of the information criteria AIC and BIC to make a choice as to which sub-model these effects should be included in and more generally selecting between the 4 alternative covariate inclusion models. Following this we present a reworking of a previously analysed dataset to illustrate the methodology and offer some guidance for the use of these models in real applications.

## Methods

### The EU model

In this work we will use the EU model of Zhou and Weinberg [4]. Following the notation of Roberts [14], an EU model each cycle *i* has a *u*_*i*_ uterine receptivity probability and each embryo *j* of cycle *i* has a survival probability *e*_*ij*_. The *u*_*i*_ and *e*_*ij*_ are represented as logistic regression submodels:

$$\mathrm{logit}\left(u_{i}\right)={\underset{˜}{\beta^{\prime }}}_{U}U_{i},$$

(1) $$\mathrm{logit}\left(e_{\mathrm{ij}}\right)={\underset{˜}{\beta^{\prime }}}_{E}E_{\mathrm{ij}},$$

where *U*_*i*_ and *E*_*ij*_ are the covariate design matrices of uterus and embryo covariates respectively, and ${\underset{˜}{\beta^{\prime }}}_{U}$ and ${\underset{˜}{\beta^{\prime }}}_{E}$ are their corresponding parameter vectors. So now the probability of a k-fold pregnancy for cycle *i* with *n*_*i*_ embryos transferred becomes:

(2) $$P_{i}^{\left(k\right)}=\left(1-u_{i}\right)\delta \left(k\right)+u_{i}P_{j=1,n_{i}}^{k}\left(e_{\mathrm{ij}}\right),$$

where *δ*(*k*) is 1 if *k* = 0 and 0 otherwise and $P_{j=1,n_{i}}^{k}\left(e_{\mathrm{ij}}\right)$ is the sum over all subsets of size *k* of the product of *e*_*ij*_ in the subset and the complement (1 − *e*_*ij*_) of the *e*_*ij*_ not in the subset. In terms of an indicator vector, s, the elements *s*_*l*_ of the vector take the values of 0 and 1 such that their sum is equal to k. So we can write

$$\begin{array}{l} P_{j=1,n_{i}}^{k}=\sum_{s\in S}\prod_{l}e_{\mathrm{ij}}^{s_{l}}{\left(1-e_{\mathrm{ij}}\right)}^{1-s_{l}}\\ S=\left\{\left(s_{1},\ldots ,s_{n}{}_{{}_{i}}\right)\in \left(0,1\right):s.t.\sum s_{l}=k\right\}. \end{array}$$

The assumptions of the EU model are that, conditional on the covariates, embryo viabilities and uterine receptivity are independent, and embryo viability is assumed independent among the embryos produced in a single IVF cycle. In this work we assume that the cycles are independent, but this assumption can be relaxed and random effects included to allow, for example, for repeat cycles from the same couples [15,16].

### Fitting the model

The model can be readily fit to data using direct maximisation of the likelihood and the details are available in [14,15]. This has been implemented in S-plus [7,17], R [14,18] and Stata [15,19]. The simulation work here used the Stata implementation, whilst the worked example was fitted using R.

### Simulation design

Simulated datasets were created assuming an EU model. The simulated datasets were designed to have similar outcomes (both in terms of pregnancy rates and twin rates) to that of the large multi-centre UK dataset from the *toward*SET project which motivated this work (see above). This simplified model only considers four covariates representing, in each sub-model, a putative prognostic variable which is to be tested in the analysis and fixed covariates which are pre-specified as being included in the analytical models:

(3) $$\begin{array}{l} \mathrm{logit}\left(u_{i}\right)=\alpha_{u}+\beta_{u}U+\beta_{\mathrm{up}}P,\\ \mathrm{logit}\left(e_{\mathrm{ij}}\right)=\alpha_{e}+\beta_{\mathrm{ep}}E_{p}+\beta_{\mathrm{ee}}E_{e}+\beta_{\mathrm{ep}2}P. \end{array}$$

We include covariates in the embryo sub-model which are measured at both the embryo and the patient level. The *U*, *E*_*p*_ and *E*_*e*_ covariates are based on the distributions of the two linear predictors from a fitted EU model in the motivating dataset:

$$U\sim N\left(-0.5,0.5^{2}\right),$$

$$E_{p}\sim N\left(-0.1,0.3^{2}\right),$$

$$E_{e}\sim N\left(3,0.6^{2}\right)$$

and have, by definition, coefficients *β*_*u*_, *β*_*ee*_, *β*_*ep*_ equal to 1. The intercept terms have values determined by the outcomes in the motivating dataset:

$$\alpha_{u}=0.1,\alpha_{e}=-3.66$$

*P* represents a new patient level covariate which is being considered for inclusion and is arbitrarily assigned a standard normal distribution *P* ~ *N*(0,1).

The final parameters *β*_*up*_ and *β*_*ep2*_ represent the effects of the putative patient variable under investigation (*P*) and a range of values are considered:

$$\beta_{\mathrm{up}}=0,0.1\ldots 1$$

$$\beta_{\mathrm{ep}2}=0,0.1,\ldots 1$$

In the case where *β*_*up*_ and *β*_*ep2*_ are zero this reproduces an overall pregnancy rate of 19.7% and an overall twin rate of 3.2% (4.6% in those cycles with two embryos transferred) close to those observed in the *toward*SET dataset. Over the range of parameters considered the overall success rate varies from 19.7 to 24.8% and the twin rate between 3.2 and 6.9%.

All combinations of the coefficients of the variables being considered (*β*_*up*_ and *β*_*ep2*_) were simulated, leading to 121 different cases. Four explicit cases are of particular interest:

1. The prognostic variable under consideration did not affect the treatment outcome (*β*_*up*_ = 0 and *β*_*ep2*_ = 0).

2. The prognostic covariate under consideration is associated with the treatment outcome at an embryo level (embryo sub-model, *β*_*up*_ = 0 and *β*_*ep2*_ = 0.1, 0.2,…,1).

3. The prognostic covariate under consideration is associated with the treatment outcome at a recipient level (uterus sub-model, *β*_*ep2*_ = 0 and *β*_*up*_ = 0.1, 0.2,…,1).

4. The prognostic covariate under consideration is associated with the treatment outcome at both levels (*β*_*up*_ = 0.1, 0.2,…,1 and *β*_*ep2*_ = 0.1, 0.2,…,1).

Simulations were performed at 5 different sample sizes, 400, 800, 1600, 3200 and 6400 treatments all with 30% single and 70% double embryo transfers, reflecting European practice.

For each simulated cycle, values for *U*, *E*_*p*_ and *E*_*e*_ are sampled from the covariate distributions. Using these values equation (3) is used to determine the probabilities *u*_*i*_ and *e*_*ij*_ and these probabilities realised by sampling from a Bernoulli distribution to determine the cycle outcome.

6000 replications were performed giving estimates with an estimated precision (half width of two-sided 95% CI) on a 5% test size of ±0.6% and ±1.3% on a 50% misclassification rate.

### Inference: Is a variable associated with outcome?

The statistical significance of the new variable measured at the patient level is tested using a likelihood ratio (lr) test comparing the full model with a reduced model excluding the tested parameter. A lr-test is used in preference to the Wald test as previous work has suggested that for these models the Wald test performs less well for small sample sizes [14]. Since the variable can be included and tested in more than one sub-model the following alternative strategies were employed and compared:

1. “Found-in-Either” strategy: Two single degree of freedom (df) lr-tests are performed, one on the model including the new variable in the uterus sub-model and another on the model including the variable in the embryo sub-model both against the reduced model that does not include the new variable. The new patient level variable is regarded as significant in this strategy if either of the lr-tests exceeds the nominal significance level.

2. “Found-in-Both” strategy: The same single df lr-test tests are performed as in Found-in-Either strategy, but both tests need to reach the nominal significance level to regard the variable as significant.

3. “Bonferroni” strategy: This strategy is the identical as the Found-in-Either strategy, but a Bonferroni correction is applied to the significance levels of the two tests to adjust for multiple testing. This strategy regards the new variable as significant at the nominal α significance level, if either of the two tests is found to be significant at an α/2 significance level.

4. “Global” strategy: A single two df lr-test is performed on the model including the new variable in both sub-models against the model not containing the variable in either sub-model. This strategy regards the new variable as significant, if the lr-test is found significant at the nominal significance level.

All hypothesis tests used the conventional P < 0.05 significance level. The test size and hence type I error rates are estimated for each scenario and compared to the nominal rates, and power estimated for the non-null cases.

For comparative purposes we also computed the estimated power based on a naive approximation where the individual sub-models were considered in isolation with the same effect size as the full EU model, a sample size based on the number of cycles and an event rate given by the pregnancy rate. This reflects a conservative power estimate (using the pregnancy rate rather than the E or U probabilities) that is potentially available from standard software, although the estimates presented here were computed using simulation.

### Model selection

Model selection is performed using Information Criteria (IC), the two IC considered here are the Akaike Information Criterion (AIC) and the Bayesian Information Criterion (BIC). The two IC are defined as

(4) $$\mathrm{AIC}=-2\ln L\left(\underset{˜}{\hat{\theta }}\right)+2p$$

(5) $$\mathrm{BIC}=-2\ln L\left(\underset{˜}{\hat{\theta }}\right)+p\ln\left(n\right)$$

where $L\left(\underset{˜}{\hat{\theta }}\right)$ is the maximum of the likelihood function, *p* is the number of parameters in the model and n is the sample size [20]. These are compared in terms of their ability to correctly determine in which sub-model the effect should be included. Note that these two IC are equivalent when the models considered have the same number of parameters (the non-nested case), as when comparing the inclusion of a variable in either of the two sub-models (embryo or uterus).

Since BIC is a sample size dependent statistic, as with other multilevel modelling situations, the sample size can be measured at any of the levels of the hierarchy [21]. In EU models the number of cycles in the dataset and the number of embryos in the dataset could both be regarded as appropriate measures of sample size. As the outcome is only observed at the patient level the BIC used here uses the number of cycles as sample size. This issue is not a concern when choosing to include a patient effect in either the uterus or the embryo sub-model (but not in both) since the BIC difference is independent of sample size.

Unlike the AIC where the difference between two models has no direct interpretation, the BIC difference can be considered as an approximation of the Bayes factor [13]. Jeffreys [12] proposed a rule of thumb for interpreting Bayes factors, and this was slightly modified by Raftery [13]. This rule of thumb is shown in Table 1. The interpretation of the BIC as a Bayes factor has

**Table 1:** **Grades of evidence of the BIC difference and the posterior probability as proposed by Raftery**[13]

**BIC difference**	**Proportion of correct classification**	**Evidence**
0-2	50-75	Weak
2-6	75-95	Positive
6-10	95-99	Strong
>10	>99	Very strong

 proved controversial, and the Raftery ’s interpretation is based on a very different scenario from the EU model. The proportion of simulations in which the correct assignment is made is estimated in each scenario and compared to the proportions suggested by Raftery.

In this work we pragmatically consider AIC and BIC as commonly applied. However it should be noted that there are subtle differences in the assumptions behind these two criteria. The AIC is designed to obtain the optimal model available assuming the true model is not one of the models considered; whereas the BIC assumes that the true model is one of the models considered [22].

## Results and discussion

### Hypothesis tests for association: type 1 error and power

**Figure 1:**
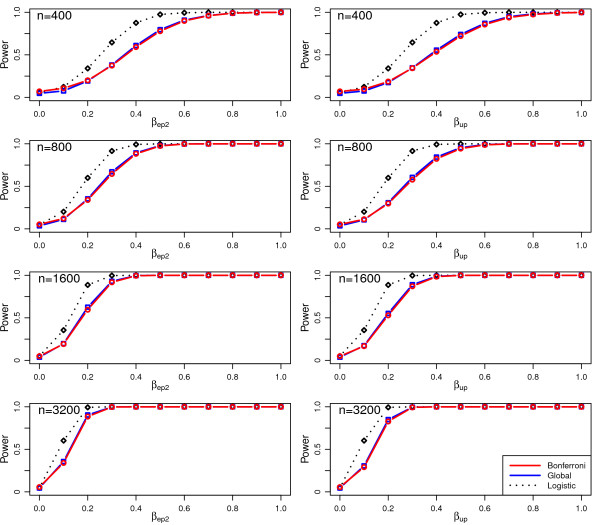
**Statistical power as a function of effect size for tests of the effect of a variable without pre-specification of the sub-model for various sample sizes.** Data is simulated for a true effect in either the embryo sub-model (left hand panels) or the uterus sub-model (right hand panels). For comparison a naive logistic power estimate is included (see text).

Table 2 show the type 1 error for the strategies investigated here. As Table 2 and Figure 1 show; the strategies which had a type 1 error close to the design test size (5%) were the Global strategy and the somewhat conservative Bonferroni strategy. The Found-in-Either strategy was found to be an overly liberal analysis with the type 1 error reaching up to 8.7%. Finally, the Found-in-Both strategy was found to be a conservative analysis having a type 1 error (2.3%-2.7%) approximately half of the designed test size. As noted previously [14], the tests were slightly more liberal for the smallest sample size.

Figure 1 shows the power to detect an effect as a function of effect size for the two strategies that have close to the nominal type I error rate (Bonferroni and Global

**Table 2:** Type 1 error rates for the strategies at the simulated sample sizes

	**Sample size**				
**Strategy**	**400**	**800**	**1600**	**3200**	**6400**
**Tests with sub-model not pre-specified**					
**Found-in-Either**	8.7%	7.4%	7.8%	8.2%	7.5%
**Found-in-Both**	2.6%	2.3%	2.3%	2.7%	2.5%
**Global**	7.0%	5.2%	4.9%	5.6%	4.9%
**Bonferroni**	4.7%	3.5%	3.8%	4.5%	3.9%
**Tests with sub-model pre-specified**					
**Uterus sub-model**	5.8%	4.9%	5.1%	5.3%	5.1%
**Embryo sub-model**	5.4%	4.7%	4.9%	5.7%	5.0%

Estimated simulation error (95% CI width) is ±0.55%.

 strategies). The two cases where the true effect is in the embryo sub-model (left hand panels) and uterus sub-model (right) are shown. Also shown in Figure 1 are naive logistic power estimates based on the component sub-models, with a probability of success equal to the birth rate. The Bonferroni strategy and the Global strategy were almost identical in terms of power, with the Bonferroni strategy being slightly more powerful for all but the smallest effect sizes. Also it is clear that a naive power calculation based on the component logistic models will severely over-estimate the power.

**Figure 2:**
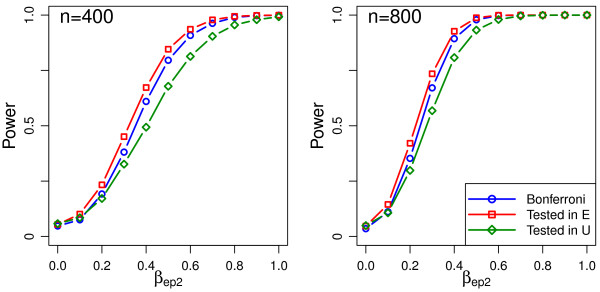
**Power curves for the scenario where the prognostic variable under consideration affects the treatment at an embryo level and is tested in either the (correct) E or (incorrect) U sub-model.** The power for a Bonferroni test with no sub-model assumption is included for comparison.

**Figure 3:**
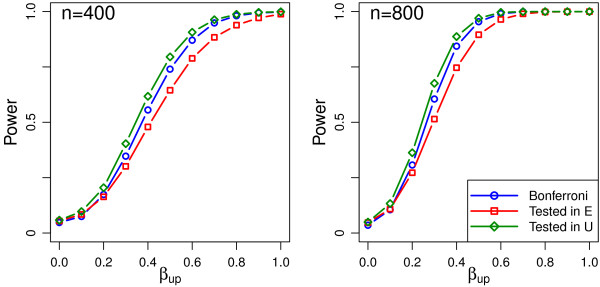
**Power curves for the scenario where the prognostic variable under consideration affects the treatment at a uterus level and is tested in either the (incorrect) E or (correct) U sub-model.** The power for a Bonferroni test with no sub-model assumption is included for comparison.

The simulations also allow us to investigate the scenario in which the sub-model is pre-specified. Table 2 shows that the type 1 error was close to the nominal level (4.9%-5.8%) when a uterus-model effect was tested in the uterus sub-model and similarly (4.7%-5.7%) when an embryo-model effect was tested in the embryo sub-model. Figures 2 and 3, show power curves when variables are correctly and incorrectly specified as acting through the embryo or uterus sub-models. The Bonferroni strategy with no assumption about the correct sub-model is presented as reference. As would be expected the power curves show that when the correct sub-model was assumed correct the power of these strategies was better than the Bonferroni strategy but if the incorrect sub-model was assumed correct then the test has less power than the Bonferroni strategy.

Thus if it is known which sub-model the effect is acting in, testing in that pre-specified sub-model can be regarded

 as an optimal strategy having a type 1 error close to the designed test size and also a higher power than it would if no such assumptions were made. However if the model specification is incorrect there is a not insignificant loss of power incurred by mis-specification and a conservative Bonferroni approach would be preferable. Since in practice even when biological evidence suggests inclusion of a patient prognostic variable in one sub-model, if the data is observational it can often be argued that the same prognostic variable may affect the other sub-model due to patient selection effects. If this may be so then the strategy of testing the variable only in the pre-specified sub-model might be advised against. For example, male infertility naturally would be included in the embryo sub-model since there is no plausible mechanism for the male to directly affect the uterine or maternal component. However as these models are usually applied to a population of infertile couples, only one of which is expected to be necessarily infertile, the fact that there is a known male cause will probably itself lead to the female partners of infertile males having greater fertility than those with fertile males.

### Sub-model selection

Firstly we consider the simple case where a variable of interest has been pre-specified and one wants to know in which of the sub-models it should be included, possibly in order to investigate mechanistic hypotheses. Tables 3 and 4 show the proportion of the correct classifications by the AIC/BIC criteria (the information criteria AIC and BIC are equivalent in this case) when selecting a single sub-model for a prognostic variable when the true effect is in just the embryo or uterus sub-model respectively. As would be

**Table 3:** AIC/BIC performance when the true model effect is in the embryo sub-model

	**Sample size**				
**Effect size (β**_**ep2**_**)**	**400**	**800**	**1600**	**3200**	**6400**
**0**	50.0%	50.1%	50.0%	51.1%	51.1%
**0.1**	53.2%	56.1%	60.4%	68.4%	76.5%
**0.2**	61.1%	68.0%	76.9%	85.5%	93.3%
**0.3**	68.7%	77.9%	86.4%	94.3%	98.5%
**0.4**	76.0%	85.6%	93.0%	98.1%	99.9%
**0.5**	81.3%	90.2%	96.9%	99.4%	100.0%
**0.6**	85.9%	94.2%	68.5%	99.9%	100.0%
**0.7**	89.1%	96.3%	99.4%	100.0%	100.0%
**0.8**	92.1%	67.7%	99.8%	100.0%	100.0%
**0.9**	94.1%	98.9%	99.9%	100.0%	100.0%
**1**	95.8%	99.5%	100.0%	100.0%	100.0%

Proportion of the simulation samples in which use of the AIC/BIC criteria would select the correct model when the true model effect is in the embryo sub-model.

 expected, the proportion of correct classifications increases from 50% (when there is no true effect) as the effect of the parameter and/or the sample size increases. Rather large sample sizes compared to typical current datasets or effect sizes are required to reliably determine the correct assignment of effect to sub-model. There is no detectable bias toward selecting either of the sub-models when there is no true effect in either model; over the range of simulation scenarios considered, the selection rates are equal at 50% to within the simulation error.

Table 5 shows the proportion of correct classifications when choosing between the sub-models according to the difference in the BIC (or equivalently AIC in this context) between the two models. The ranges and expected

**Table 4:** AIC/BIC performance when the correct model when the true model effect is in the uterus sub-model

	**Sample size**				
**Effect size (β**_**up**_)	**400**	**800**	**1600**	**3200**	**6400**
**0**	50.0%	49.9%	50.0%	48.9%	48.9%
**0.1**	52.0%	55.5%	59.9%	66.3%	74.4%
**0.2**	58.0%	65.3%	73.7%	83.1%	91.3%
**0.3**	64.7%	73.4%	83.5%	91.2%	97.6%
**0.4**	70.9%	80.7%	89.8%	96.2%	99.3%
**0.5**	74.8%	85.2%	94.1%	98.4%	99.9%
**0.6**	78.5%	89.0%	96.4%	99.4%	100.0%
**0.7**	81.4%	91.5%	97.6%	99.7%	100.0%
**0.8**	83.9%	93.5%	98.5%	99.9%	100.0%
**0.9**	86.1%	94.8%	99.2%	100.0%	100.0%
**1**	88.1%	95.9%	99.4%	100.0%	100.0%

Proportion of the simulation samples in which use of the AIC/BIC criteria would select the correct model when the true model effect is in the uterus sub-model.

**Table 5:** Assignment to E or U sub-model in the simulation study

**AIC/BIC difference**	**Expected (Raftery’s)**	**Sample size**				
		**400**	**800**	**1600**	**3200**	**6400**
**0-2**	50-75%	59.3%	60.4%	61.1%	62.9%	64.9%
**2-6**	75-95%	82.3%	85.4%	86.4%	86.5%	87.1%
**6-10**	95-99%	96.2%	97.3%	97.8%	97.5%	97.9%
**>10**	>99%	99.4%	99.8%	99.2%	100.0%	100.0%

Proportion of correct classifications using the AIC/BIC in terms of the observed BIC difference between models. The proportions shown are averaged over all the scenarios considered in Tables 3 and 4. The expected proportions of Raftery are also listed for these differences as a reference.

 proportion of true classifications using the ranges from Raftery [13] are shown for comparison. Despite the very different application here from the original work, the proportions of correct model choices are in good agreement with Raftery, and differences in BIC between models of ≥6 are required to provide strong evidence (>95% probability of correct assignment) in favour of one or the other sub-model. The previously suggested criterion of an AIC difference of >2 [5] corresponds to what Raftery termed “positive evidence” and a probability of >75% that the assignment is correct. From the table we also observe that the BIC differences proportions of correct classifications increases slightly as the sample size increases, this may be due to the fact that BIC is a large sample Bayes factor approximation and as the sample size increases the estimate becomes closer to the true Bayes factor estimate [13].

**Figure 4:**
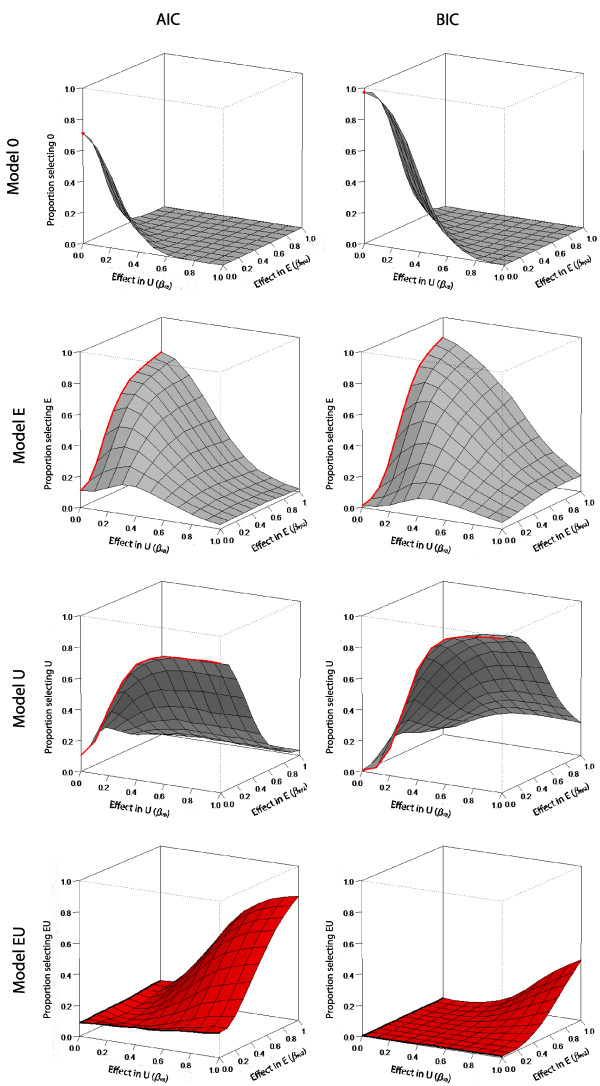
**The proportions of a patient variable assigned to each of the four alternative models using the AIC (left hand panels) or BIC (right hand panels).** Data shown is for a sample size of 800. The red point/lines/shading indicate the regions corresponding to the true model and the gray lines/shading indicate the model is incorrect. The “true” models include the covariate in neither (model 0), the E (model E), U (model U) or both (model EU) sub-models as indicated.

A more realistic case is that where a variable is being considered for inclusion in a model, maybe for prognostic or predictive purposes, and there is no evidence as to which sub-model, if any it should be included in. Thus there are four possible models to be considered: omit the variable, include it in E, include it in U or include it in both sub-models. Figure 4 shows, for an illustrative sample size of 800, the proportion of simulated datasets which are assigned to the 4 potential models as a function of the effect sizes in each of the models using the AIC (left hand panels) or BIC (right hand panels). As with other applications [23,24], the AIC is more likely to choose a larger model than the BIC but conversely is less able to correctly identify a true effect. However, as can be seen from the margins in the lower panels of Figure 4, when the true effect is in only one of the sub-models the AIC selects the model that includes the covariate in both sub-models 11-15% of the times (with the larger misclassification rates observed on larger sample sizes), whereas the BIC incorrectly selects the larger model <1% of the times. Both AIC and BIC become more likely to select the correct model as the sample size increases (data not shown).

Tables 3 and 4 and Figure 4 also indicate that, for the same effect size, if the information criteria are used for

 model selection then it is slightly more likely that a prognostic variable will be correctly included if it truly acts through the embryo sub-model than if it belongs in the uterus sub-model. This reflects the greater amount of information available at the embryo level compared to that at the patient level.

### Worked example

As the dataset was large, all the potential factors were included in the model. To determine in which of the sub-models (E, U or both) a factor would be included in a mixture of pre-specification based on other work and selection on the basis of AIC was used. A simple likelihood-ratio test comparing the fitted model with a reduced model with that parameter excluded was used to give an indicative P-value for each factor, acknowledging that this test does not allow for the model selection process. Although the AIC allowed statements to be made as to the best fitting model and therefore the best supported mechanism (E or U) for the mediation of the effects, that work struggled to provide any indication as to the strength of the evidence and noted the need for further research.

Table 6 shows the AICs for the fitted model where each factor in turn is permuted to be in E, U, both or neither sub-model whilst the remaining variables are specified as per the final selected model. Age was pre-specified to act through both E and U as previous data from donated eggs suggests that both maternal age and egg age are prognostic, although the AIC would suggest that it should only be included in the E sub-model. Centre and year as proxies for treatment and population changes were also included in both models. Several factors are included which would not be selected by AIC.

Whilst we would not necessarily advocate formal hypothesis tests for prognostic factors in such a large dataset, preferring to focus on effect sizes, nevertheless such tests were offered in the original work. The possible tests comparing each factor in turn with a null model for that factor, holding all other factors at their selected locations and performing a 1 or 2df likelihood ratio test are shown in Table 7. The highlighted values indicate the P-values (conditional on model selection) used in the original analysis,

The Bonferroni approach would give identical results at a critical value of P < 0.025 for the E and U tests, but we note that the test used previously does over-state the significance. For transfer day the P = 0.024 would be considered borderline significant with the recommended method. We note also in this example with a large sample size, that the 2df test of E + U gives overall similar conclusions, but that the significance levels from the two 1df Bonferroni test and the 2df global tests can diverge appreciably (eg Attempt number). As always, there is no substitute for carefully formed pre-specified hypotheses.

With regard to the interpretation of the assignment of effects to the E or U sub-model, the previous work tentatively used an arbitrary AIC difference of >2 to make statements that there was reasonable evidence that one mechanism should be preferred. The work here suggests that this statement is perhaps too optimistic, with a misclassification rate of ~25% and to make strong statements AIC differences of ⪆6 are required. If this more stringent level were used, only one of the five ascribed

**Table 6:** AIC (difference from null model) for the motivating dataset

**Variable**	**AIC**			
	**null**	**E**	**U**	**E + U**
Number of embryos transferred	0	**−0.6**	1.1	−0.1
Age group	0	**−178.2**	−153.8	−175.1
Number of embryos created	0	−13.7	**−19.3**	−9.3
IVF Attempt number	0	−4.3	**−8.1**	−2.6
ICSI^1^	0	**0.5**	1.6	2.4
Pregnancy History^2^	0	−2.6	**−8.5**	−3.1
Duration infertile	0	−1.7	**−10.4**	−3.0
Tubal diagnosis	0	−9.9	**−14.2**	−12.8
PCO^3^ diagnosis	0	2.0	**2.0**	3.9
Endometriosis	0	0.1	**−1.2**	0.8
Idiopathic diagnosis	0	1.8	**1.6**	2.4
Male diagnosis	0	**0.6**	1.3	2.6
Donor sperm	0	**2.0**	0.8	1.1
Transfer day^4^	0	**−3.5**	−1.9	−1.6
Year	0	−8.0	−11.2	**−13.0**
Treatment Centre	0	−11.2	−9.2	**−24.7**

^1^Intercytoplasmic sperm injection, a variation on standard IVF.

^2^A composite variable indicating the number and type of previous pregnancies.

^3^Polycystic Ovary Syndrome.

^4^Embryos were cultured for 2 or 3 days before transfer to the potential mother.

AIC (difference from null model) for models for the motivating dataset in which each included variable is assigned to either none (null), the embryo (E), uterus (U) or both (E + U) sub-models. The highlighted entries indicate the assignment in the final selected model, usually (but not in all cases – see text) that with the lowest AIC.

**Table 7:** Significance tests for the motivating dataset

	**P-values**		
**Variable**	**E**	**U**	**E + U**
Number of embryos transferred	**0.10**	0.24	0.088
Age group	**<0.001**	<0.001	<0.001
Number of embryos created	<0.001	**<0.001**	0.001
IVF Attempt number	0.016	**0.003**	0.024
ICSI	**0.22**	0.53	0.45
Pregnancy History	0.035	**0.002**	0.019
Duration infertile	0.033	**0.001**	0.008
Tubal diagnosis	0.001	**<0.001**	<0.001
PCO diagnosis	1.00	**1.00**	0.95
Endometriosis	0.17	**0.074**	0.20
Idiopathic diagnosis	0.66	**0.53**	0.45
Male diagnosis	**0.24**	0.40	0.50
Donor sperm	**1.000**	0.27	0.24
Transfer day	**0.024**	0.052	0.048
Treatment Year	0.003	0.001	**<0.001**
Treatment Centre	0.001	0.002	**<0.001**

P-values from formal hypothesis tests for adding each variable to the model in either the E, U or both (E + U) sub-models. Tests are 1 or 2 degree of freedom likelihood ratio tests. Highlighted values indicate the selected final model. See Table 6 for more information on the variables.

 assignments would be considered to have strong support. This underlines the fact that there is very limited statistical power to determine these assignments.

Finally we note that the earlier work observed that the year and centre effects, presumed surrogates for variation in clinical practice and population not captured by the available covariates, were difficult to interpret. If, rather than AIC, the BIC criteria were used and the model selected accordingly, then these effects would not be included. This suggests that these complex effects could be considered as resulting from over fitting in the large dataset.

### Guidance for practice

EU models due to their biological derivation can in principle separate the effect of the embryo’s viability and the receptivity of the uterus, and yield statistical models with a causal interpretation. Even if the mechanistic basis is over-simplified or the data subject to confounding, the models have proved rich enough to be of practical utility whilst solving the statistical issues of partial-observability.

When attempting to determine whether a variable is associated with outcome, there will often be no prior evidence from which one can pre-specify in which sub-model a variable of interest should be included. The simulations indicate that for practical purposes a reasonable strategy would be to perform two single df likelihood ratio tests; one for the model including the variable in the uterus sub-model and one for the model which includes the variable in the embryo sub-model. The variable is then considered to be statistically significant if either of the lr-tests is found to be significant at half the nominal level. The commonly used strategy of testing the effect in both sub-models without the Bonferroni correction leads to an over-liberal test. Over the range of parameters and models simulated the concern that this approach may be over-conservative due to the tests being non-independent is not borne out.

If prior knowledge reliably allows the pre-specification of the sub-model, then a single test at the nominal level should be preferred. However if this model is incorrectly specified then this test has less power than the Bonferroni approach with no assumptions as to the sub-model: indeed the loss from miss-specification exceeds the gain from correct specification. The strong patient selection effects in most observational IVF datasets mean that *a priori* assumptions based on biological and clinical arguments can be misleading. Thus unless the evidence for an appropriate sub-model is strong a conservative approach of testing in both models with a Bonferroni correction would be advised.

The second major application of these models has been in the development of prognostic models of patient outcomes. When a patient-level variable is to be included, the AIC and BIC can be used to make the distinction in which sub-model to include that variable. The criteria suggested by Raftery [13] and summarised in Table 1 provide a good basis for assessing the weight of evidence for the resultant assignment. When considering including a patient level prognostic variable in an EU model there is a need to select between the 4 possible alternative models; BIC seems to perform well with the number of patients being used as the sample size parameter*.* However large sample sizes are required to determine the assignments with any degree of reliability and so to draw causal inferences from the assignment.

To date EU models have only been used to analyze observational datasets (not clinical trials) and there is little guidance as how such studies can be adequately powered, with most studies relying on heuristic or feasibility arguments. The power curves and the percentages of correct classifications presented here can be used to aid in the design of future studies.

### Extensions to the EU model

In this paper we investigated, using simulation, the performance of various strategies for model selection and significance testing for a patient prognostic variable in an EU model. Whilst these simulations inevitably cover only a small subset of the parameter space, they do, we believe, provide insight as to the performance of the EU model and guidance as to its use for practical data analysis.

The work here has focussed on the case where there is only a single treatment for each couple and has not considered the potentially complex hierarchical structure that can be introduced in real data with repeat treatments. The EU model can in principal be extended to include such correlations, and the nested cases with random intercepts in the E and U submodels have been considered elsewhere [15,16]. Simulation studies with these extended models are not yet computationally feasible. The work so far suggests that in practical applications including random effects has a negligible impact on the fixed effect estimates and inference: although the variance estimates are useful in themselves, model selection and inference for fixed effect parameters may be adequately performed whilst ignoring the higher level structure.

The major caveat is the underlying assumption in the simulation work that the EU model does reflect the true behaviour and further work is required to relax this assumption. Earlier work [14] included some additional sources of variability and in that case no qualitatively different behaviour was observed. A collaborative model has been proposed [25], but to date does not consider covariates and its properties are not yet well understood. Although the simplistic mechanistic basis of the EU model can be contested and practical interpretations have gone beyond the putative mechanism, the multi-level structure does reflect the structure of the data and account for the major statistical issue of the partial observability of the outcome. The strong assumptions of conditional independence between embryos and between the embryo and uterus effects do need to be acknowledged and further work is required to determine the extent to which the model estimates and their interpretation depend on these assumptions. For datasets with one cycle per patient, additional correlations will be indistinguishable from those induced by additional covariates and as noted above approaching these questions using simulation is as yet infeasible. Real datasets with multiple cycles per patient are subject to strong selection biases as a complex interaction between health care provider policy, patient choice and resource availability will determine of the number of cycles as well as the outcomes of previous cycles. Given that the EU model (particularly if patient random effects are included) has a rich structure which can accommodate a range of potential correlations, and that real datasets have only limited numbers of repeat cycles, it is unlikely that any realistic dataset will have sufficient power to identify departures from the EU model.

## Conclusion

We believe that the EU model approach, despite its limitations, is currently the only practical approach that can properly account for the data structure encountered in the analysis of IVF data with multiple embryo transfer. There is now a sufficient body of methodological and practical work to support its more widespread use in real applications.

## Competing interests

The authors declare they have no competing interests.

## Authors’ contributions

CS designed and performed the simulation work. AP and SR supervised this work. SR conceived the project and added the worked example. All authors contributed to the writing of the manuscript. All authors read and approved the final manuscript.

## Pre-publication history

The pre-publication history for this paper can be accessed here:

http://www.biomedcentral.com/1471-2288/13/73/prepub
